# Prostate‐specific membrane antigen expression in the vasculature of primary lung carcinomas associates with faster metastatic dissemination to the brain

**DOI:** 10.1111/jcmm.15350

**Published:** 2020-05-11

**Authors:** Jayendrakishore Tanjore Ramanathan, Suvi Lehtipuro, Harri Sihto, József Tóvári, Lilla Reiniger, Vanda Téglási, Judit Moldvay, Matti Nykter, Hannu Haapasalo, Vadim Le Joncour, Pirjo Laakkonen

**Affiliations:** ^1^ Translational Cancer Medicine Research Program Faculty of Medicine University of Helsinki Helsinki Finland; ^2^ Faculty of Medicine and Health Technology Tampere University Tampere Finland; ^3^ Clinicum Faculty of Medicine University of Helsinki Helsinki Finland; ^4^ Department of Experimental Pharmacology National Institute of Oncology Budapest Hungary; ^5^ SE‐NAP Brain Metastasis Research group 2nd Department of Pathology Semmelweis University Budapest Hungary; ^6^ 1st Department of Pathology and Experimental Cancer Research Semmelweis University Budapest Hungary; ^7^ Department of Tumor Biology National Korányi Institute of Pulmonology–Semmelweis University Budapest Hungary; ^8^ Science Center Tampere University Hospital Tampere Finland; ^9^ Department of Pathology University of Tampere and Fimlab laboratories Tampere Finland; ^10^ Laboratory Animal Centre HiLIFE – Helsinki Institute of Life Science University of Helsinki Helsinki Finland

**Keywords:** brain metastasis, glioblastoma, proliferating microvasculature, PSMA

## Abstract

Glioblastomas and brain metastases (BM) of solid tumours are the most common central nervous system neoplasms associated with very unfavourable prognosis. In this study, we report the association of prostate‐specific membrane antigen (PSMA) with various clinical parameters in a large cohort of primary and secondary brain tumours. A tissue microarray containing 371 cases of ascending grades of gliomas pertaining to astrocytic origin and samples of 52 cases of primary lung carcinomas with matching BM with follow‐up time accounting to 10.4 years was evaluated for PSMA expression using immunohistochemistry. In addition, PSMA expression was studied in BM arising from melanomas and breast carcinomas. Neovascular expression of PSMA was evident alongside with high expression in the proliferating microvasculature of glioblastomas when compared to the tumour cell expression. This result correlated with the results obtained from the *in silico* (cancer genome databases) analyses. In gliomas, only the vascular expression of PSMA associated with poor overall survival but not the tumour cell expression. In the matched primary lung cancers and their BM (n = 52), vascular PSMA expression in primary tumours associated with significantly accelerated metastatic dissemination to the brain with a tendency towards poor overall survival. Taken together, we report that the vascular expression of PSMA in the primary and secondary brain tumours globally associates with the malignant progression and poor outcome of the patients.

## 1 INTRODUCTION

Gliomas, neoplasms of glial cell origin, constitute approximately 30% of all nervous system tumours and 80% of all malignant brain tumours.^1^ Glioblastoma, a grade IV astrocytoma, is the most frequent, aggressive and lethal glioma.^2^ Glioblastomas harbour a dense abnormal vasculature, display large hypoxic and necrotic areas and contain extensively proliferating tumour cells with the intrinsic ability to disseminate and colonize the organ far beyond the principal tumour mass. The current standard of care comprising surgery followed by radio‐ and chemotherapy provides only a modest improvement in the progression‐free and overall survival, and patient prognosis remains dismal.^3^, ^4^ Brain metastases (BM) of solid tumours are the most frequent intracranial tumours and about 10‐fold more common than primary brain tumours.^5^ BMs are associated with severe neurological symptoms and abysmal prognosis compared with other metastatic sites.^6^ The primary tumours most often responsible for BM are melanomas (5%‐20%), and lung (36%‐64%) and breast cancers (15%‐25%).^7^

Prostate‐specific membrane antigen (hereon referred to as PSMA) also known as glutamate carboxypeptidase II (GCP II), N‐acetyl‐L‐aspartyl‐L‐glutamate peptidase I (NAALDase I) or N‐acetyl‐aspartyl‐glutamate (NAAG) peptidase is a transmembrane glycoprotein encoded by the *FOLH1* gene. Until today, only two well‐defined physiological roles of PSMA are known: the folate‐hydrolysing activity in the small intestine and the NAAG‐hydrolysing activity in the nervous system.^8^, ^9^ PSMA is expressed in tumour cells of almost all prostate cancers, and its increased expression is associated with tumour aggressiveness, metastasis and recurrence.^10^, ^11^ Numerous studies have shown that the normal vasculature is devoid of PSMA expression, while tumour neovasculature often shows high PSMA expression.^12^, ^13^, ^14^ Moreover, PSMA expression has been reported in benign gliomas (grade I), malignant glioblastomas (grade IV) and breast cancer BM with very limited number of patients.^15^ Conway *et al* ^16^ showed that PSMA expression is critical during angiogenesis as PSMA null mice were unable to mount a pathological angiogenic response.^16^ In addition, Grant et al reported that PSMA‐regulated angiogenesis was independent of VEGF by using a mouse model of oxygen‐induced retinopathy.^17^

Our study is in line with the previous reports about PSMA expression in the tumour neovasculature. However, we accompany our immunohistochemical study with the PSMA mRNA level expression analyses in large genetic data sets. We report higher PSMA expression in glioblastomas compared to lower‐grade gliomas. For the first time, we report PSMA expression in the proliferating microvasculature (PM) of glioblastomas, which is one of the histopathological features that differentiate glioblastoma from the lower‐grade gliomas. Moreover, only the vascular PSMA expression in gliomas is associated with poor overall survival. We also had an exceptional opportunity to screen rarely available specimens of the matched primary lung cancers and their BM as well as samples from melanoma and breast cancer BM. Importantly, PSMA expression in the primary lung tumour vasculature is associated with significantly accelerated metastatic dissemination to the brain with a tendency towards poor overall survival. Overall, the large sample size along with the *in silico* data analyses provides more comprehensive view on the role of PSMA compared with prior studies involving much smaller patient cohorts.

## 2 MATERIALS AND METHODS

### 2.1 Patient material

#### 2.1.1 Gliomas

The ethical committee of Tampere University Hospital and the National Authority for Medicolegal Affairs in Finland approved the usage of tumour tissue array and the study design. The tumour samples were collected from the patients who underwent surgery at the Tampere University Hospital during the years 1983‐2009. The astrocytoma specimens were initially fixed in 4% phosphate‐buffered formaldehyde and then processed into paraffin blocks. One histologically representative tumour region was selected from each specimen. From the selected regions, 0.6‐mm tissue cores were mounted into tissue microarray (TMA) blocks.^18^ Our tumour series included 371 gliomas of astrocytic origin ranging from 41 (11.05%) grade I pilocytic astrocytomas, 46 (12.39%) grade II diffuse astrocytomas, 25 (6.73%) grade III anaplastic astrocytomas and 259 (69.81%) grade IV glioblastomas (Table 1) based on the diagnosis at the time of the surgery. Of these 371 gliomas, 288 were primary and 83 were recurrent tumours from 174 men and 114 women (Table 1).

**TABLE 1:** Clinicopathologic features of astrocytomas

Astrocytoma	Grade I	Grade II	Grade III	Grade IV	Total
No. of patients^a^	41	46	25	259	371
Primary	33	34	16	205	288
Residual					
Second	7	11	6	43	67
Third‐sixth	1	1	3	11	16
Sex (n)^b^					
Male	20	24	8	122	174
Female	13	10	8	83	114
Age (y)^b^					
Median	11	40	39.50	62.00	—
Mean ± SD	18.4 ± 16.7	42.8 ± 17.5	42.2 ± 14.5	61.4 ± 11.0	—
Minimum	1	12	23	4	—
Maximum	57	83	81	84	—
Follow‐up					
End survivors (n)	26	13	5	7	51
5‐y survival (%)	81.3	38.2	31.3	3.4	17.8

^a^ Includes primary and secondary tumours.

^b^ Includes only primary tumours.

#### 2.1.2 Primary lung tumours and BMs

Samples of surgically resected, paired primary lung carcinomas and their associated BMs from 52 patients, as well as breast carcinoma BMs from 18 patients and melanoma BMs from 19 patients, were analysed in this study. Additionally, TMAs consisted of primary lung carcinomas from 3 patients and lung BMs from 4 patients apart from the paired samples. Permissions to use the archived tissues have been obtained from the Local Ethical Committee of the Semmelweis University (Budapest, Hungary) (TUKEB‐1552012, TUKEB‐5102013 and TUKEB‐862015), and the study was performed in compliance with the Declaration of Helsinki. The samples were fixed in 10% neutral‐buffered formalin, dehydrated and cleared in increasing concentration of ethanol and xylol before embedding into IHC‐grade paraffin. For the TMA, haematoxylin and eosin‐stained sections were used to define the tumour areas and three representative 2‐mm cores were obtained from each case and inserted in a grid pattern into a recipient paraffin block using a tissue arrayer (3DHISTECH, Budapest, Hungary).

### 2.2 Immunohistochemistry

Specificity of the anti‐PSMA antibody was verified using samples from normal human liver and kidney that showed labelling of hepatocytes and renal tubules as anticipated (Figure S1A,B). In addition, PSMA protein level was verified using extracts from the LnCAP (PSMA‐positive) and PC3 (PSMA‐negative) prostate cancer cell lines (Figure S1C) to confirm the detection of the expected PSMA protein band (84 kD).

The PSMA and CD31 expression was detected from the sections by using the TSA Kit (PerkinElmer) according to the manufacturer's instructions. Tumour samples were deparaffinized by immersing the slides in xylene and rehydrated with graded ethanol (100%‐70%) and water. Briefly, the antigen was retrieved with heat treatment in citrate buffer (1.8 mmol/L citric acid, 8.2 mmol/L sodium citrate, pH 5.0). The endogenous peroxidase activity was irreversibly inactivated by incubating the sections with 3% H_2_O_2_‐MetOH for 10 minutes and blocked with TNB buffer (0.1 mol/L Tris‐HCl (pH 7.5), 0.15M NaCl and 0.5% blocking reagent from TSA kit) for 30 minutes. The primary antibody, rabbit anti‐human mAb for PSMA (ab133579, Abcam, 4 µg/mL) and mouse anti‐human CD31 (M0823, DAKO), was applied overnight at 4°C. After primary antibody incubation and washes with TNT (Tris/NaCl/Tween‐20) buffer, the sections were incubated with the biotinylated secondary antibody (goat anti‐rabbit (E0432) and goat antimouse (E0433), Dako) for 60 minutes. The signal was amplified by using the SA‐HRP and biotinylated tyramide and finally visualized using the AEC (0.95M 3‐amino‐9‐ethylcarbazole, 6% N,N dimethylformamide, 94% sodium acetate, 0.01% H_2_O_2_). The sections were counterstained with Mayer's haematoxylin.

### 2.3 Evaluation of immunostaining and statistical analysis

Overall analysis was performed for all patients whose tumour tissue was available for immunohistochemistry. PSMA‐stained sections were scanned using the 3DHISTECH Pannoramic 250 FLASH II digital slide scanner at an absolute magnification of 20×. The evaluation of the scanned sections was performed blinded to the clinical data. The PSMA staining in the TMAs was scored as negative (0), weakly positive (1), moderately positive (2) or highly positive (3) and then grouped as negative (0) or positive (1‐3 = 1) for further statistical analysis (Figure S2). We graded separately the vascular and cellular expression of PSMA and associated it with the clinical and molecular pathological parameters. The samples in the TMA that lacked vascular structures were marked as empty and excluded from the analysis. An experienced neuropathologist (HH) verified the PSMA expression in the tumour cells and in the vasculature. Overall survival was calculated from the date of primary tumour diagnosis to the date of death, censoring the patients who were alive at the end of the follow‐up period, and the progression‐free survival from the date of diagnosis to the date of tumour progression or death, censoring patients without detected progression or death. Brain metastasis‐free survival was calculated from the date of primary tumour diagnosis to the date of brain surgery. Brain metastasis was reported in all patients in the cohort. The statistical analysis was performed using the IBM SPSS statistics 21.0 software for Windows. Chi‐squared and non‐parametric tests were used to analyse the associations between PSMA expression and various clinical and molecular pathological parameters. Overall, progression‐free and brain metastasis‐free survival were analysed using the Kaplan‐Meier and survival between the groups was compared using the log‐rank and Breslow tests. The overall survival in primary lung carcinomas was analysed in 55 patients including both the paired (n = 52) and unpaired (n = 3) samples. The analysis of time to metastatic dissemination was performed using only the paired (n = 52) patient samples. A *P*‐value of <0.05 was considered significant. Statistical analyses for public data sets were performed using the R version 3.2.2.

### 2.4 Tumour data sets

The clinical data and level 3 mRNA microarray data (Agilent 244K The Cancer Genome Atlas [TCGA] Custom 1‐2) of primary glioblastoma (GBM) samples were downloaded from the NCI Genomic Data Commons. Combining multiple samples from the same patient using median and removing samples without clinical data resulted in 567 samples. FPKM values for the GBM anatomic structures (122 samples) and sample information were downloaded from the Ivy GAP website (http://glioblastoma.alleninstitute.org/).^19^ Data were log2‐transformed. We have used TCGA nomenclature for the glioblastoma terminology.

## 3 RESULTS

### 3.1 PSMA is highly expressed in the hyperplastic proliferating microvasculature of grade IV gliomas

**FIGURE 1:**
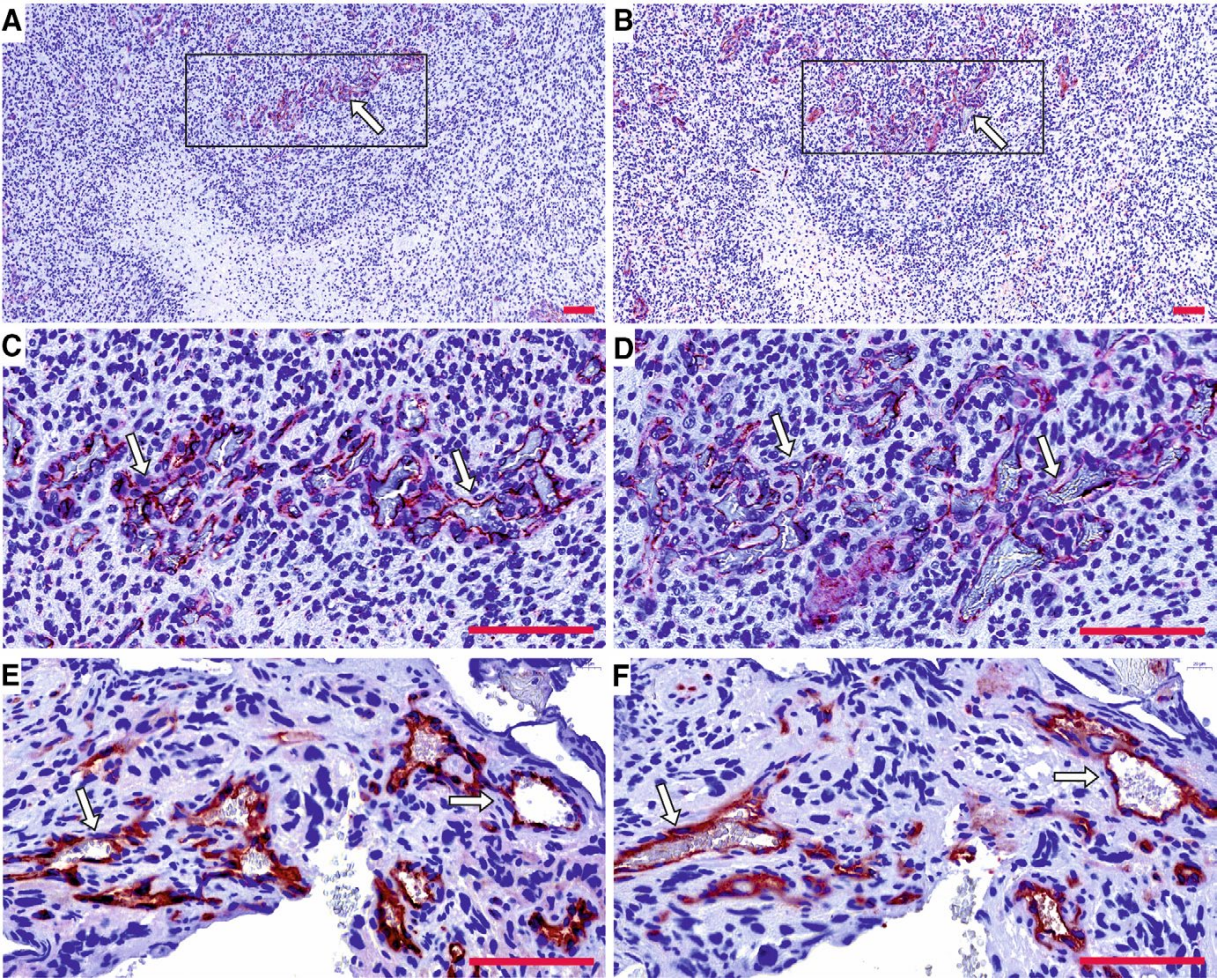
Expression of the prostate‐specific membrane antigen (PSMA) in gliomas. PSMA expression in the vasculature (A, C, E) of grade IV glioblastoma was validated by staining the blood vessels using anti‐CD31 antibody (B, D, E) in a subsequent tissue section. Arrows point the high PSMA (A, C) and CD31 expression (B, D) in the hyperplastic proliferating microvasculature. A and B, magnification 10x; C and D, higher magnification of the boxed area in A and B; E and F, high magnification micrograph. Scale bar = 100 μm

To evaluate the PSMA protein expression in human glioma samples, TMAs containing 371 grade I‐IV gliomas were stained using the anti‐PSMA antibody. Vascular expression of PSMA (Figure 1A,C,E) was validated by visualizing the blood vessels using an anti‐CD31 antibody (Figure 1B,D,F). We grouped the samples as positive (1) or negative (0). For the vascular expression, we excluded the samples that did not show any vascular structures in the TMA samples. PSMA expression was most frequently detected in the vasculature of glioblastomas (30.8%, 74/166) (*P* = 0.044) (Table 2) highlighting the distinct angiogenic feature of glioblastomas. The less angiogenic, lower‐grade gliomas showed reduced PSMA expression in their vasculature: grade III (14.2%, 3/18), grade II (17.9%, 7/32) and grade I (15%, 6/34) (Table 2). We next analysed PSMA expression in tumour cells to address whether an association exists between the vascular and cellular expression of PSMA. About 1/3 of the grade I (31.7%, 13/28) and II tumours (32.6%, 15/31) showed PSMA expression in tumour cells, whereas 56% (14/11) of grade III and 44.7% (116/143) of grade IV gliomas (Table 2) expressed PSMA in tumour cells. However, the overall expression in tumour cells was lower than the one detected in the tumour blood vessels.

**TABLE 2:** Histological classification of astrocytic tumours based on PSMA expression

Tumour grade		Pilocytic grade I	Diffuse grade II	Anaplastic grade III	Glioblastoma grade IV	Total	*P*‐value chi‐square
PSMA (+/−)	Vascular	15% (6/34)	17.9% (7/32)	14.2% (3/18)	30.8% (74/166)	26.47% (90/250)	**.044**
PSMA (+/−)	Tumour cells	31.7% (13/28)	32.6% (15/31)	56% (14/11)	44.7% (116/143)	42.5% (158/213)	.102

Abbreviation: PSMA, prostate‐specific membrane antigen.

**FIGURE 2:**
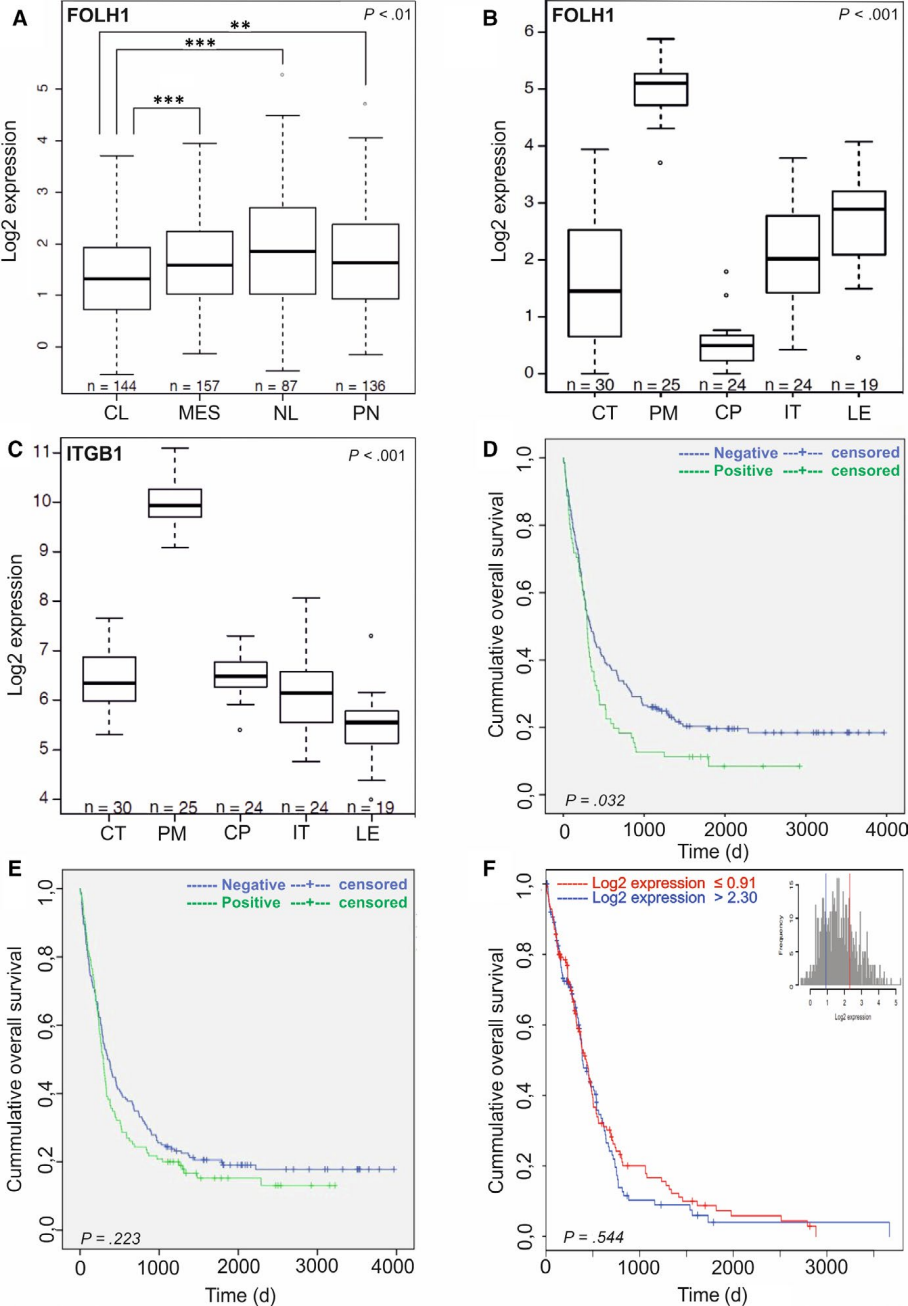
Analysis of PSMA/*FOLH1* mRNA expression using the TCGA database and association of PSMA/*FOLH1* expression with patient survival in gliomas. A, Expression of PSMA/*FOLH1* was lower in the classical subtype (CL) compared to the other subtypes in the TCGA analysis (*P* < 0.01). B, The proliferating microvasculature (PM) showed the highest and the cellular pseudopalisades (CP) the lowest PSMA expression when compared to other groups in the Ivy GAP database analysis (*P* < 0.001). C, Integrin β1 is specifically up‐regulated in the proliferating microvasculature (PM) in the Ivy GAP database analysis similar to the PSMA/*FOLH1* expression. D, The vascular PSMA expression is associated with poor overall survival of the patients (n = 263, *P* = 0.032). E, No significant association with the overall survival was observed when the tumour cell expression of PSMA protein was analysed (n = 287, *P* = 0.223). F, No significant association with the overall survival was observed when PSMA mRNA expression was analysed using the TCGA database (n = 567, *P* = 0.544). PSMA, prostate‐specific membrane antigen; TCGA, The Cancer Genome Atlas

One of the diagnostic histopathological features that differentiate glioblastoma from the lower‐grade gliomas is the presence of proliferating hyperplastic microvasculature that appears as tufted glomeruloid bodies and exists adjacent to the necrotic foci with bordering pseudopalisading cells. The glomeruloid tuft‐like structures in certain tumour samples in the TMAs were positive for PSMA. Therefore, we analysed the entire tumour block of two grade IV glioblastomas that were highly positive for the vascular expression of PSMA. This analysis confirmed that not the pre‐existing mature vessels, but the proliferating hyperplastic microvasculature expressed high levels of both PSMA (Figure 1A,C,E) and a vascular marker CD31 (Figure 1B,D,F). To study PSMA (*FOLH1*) expression in the glioblastoma subtypes, we analysed 567 mRNA microarray samples using the TCGA GBM data set (see Materials and Methods, and nomenclature from the TCGA). When we compared the expression of PSMA between the different subtypes, we observed decreased PSMA expression in the classical (CL) subtype compared to the other subtypes (*P* < 0.01, Wilcoxon rank‐sum test) (Figure 2A). Therefore, decreased PSMA expression might be associated with the classical subtype.

Next, we analysed PSMA (*FOLH1*) expression using the Ivy GAP RNA‐Seq data set (http://glioblastoma.alleninstitute.org/) that, in contrast to other cancer genome data sets, shows the individual gene expression profiles for the different anatomic structures, such as leading edge (LE), infiltrating tumour (IT), cellular tumour (CT), PM and cellular pseudopalisades (CP), which were isolated by using the laser microdissection.^19^ Consistent with our findings, PM showed the highest (*P* < 0.001, Wilcoxon rank‐sum test) and the CP the lowest PSMA expression when compared to other groups (*P* < 0.001, Wilcoxon rank‐sum test) (Figure 2B). As it has been reported that PSMA is involved in angiogenesis by activating the laminin‐mediated integrin β1 (ITGB1) signalling,^16^ we analysed the *ITGB1* expression using the Ivy GAP. Also, in this data set, *ITGB1*was up‐regulated in the PM when compared with all the other anatomic structures (Figure 2C) (*P* < 0.001).

### 3.2 Vascular expression of PSMA associates with poor prognosis in gliomas

We next analysed the association of PSMA protein level with patient survival in our TMA material using the log‐rank test. The vascular PSMA expression was associated with poor overall survival of patients (n = 263) (*P* = 0.032, log‐rank test) (Figure 2D and Table 3), whereas no association with survival of PSMA in tumour cells was detected (n = 287) (*P* = 0.223, log‐rank test) (Figure 2E and Table 3). We also analysed the association of PSMA (*FOLH1)* expression with patient survival using the TCGA GBM data set (n = 567 mRNA microarray samples), which contains transcriptome data of the whole tumour bulk comprising mostly of tumour cells. No association with patient survival was observed when patients were separated based on the PSMA (*FOLH1)* expression as the highest 25% compared to the lowest 25% (*P* = 0.544, log‐rank test) (Figure 2F). Thus, PSMA expression in the vasculature of gliomas associates with poor overall survival while no significant association with patient survival of PSMA expression in tumour cells was detected.

**TABLE 3:** Overall survival in gliomas based on PSMA expression

Tumour	Features	Function	PSMA (+) Total/censored	PSMA (−) Total/censored	Total Total/censored	*P*‐Value Log‐rank
Gliomas Grade (I‐IV)	Vascular	OS	71/7	192/39	263/46	**.032**
	Tumour cells	OS	115/18	172/33	287/51	.223

Abbreviations: OS, overall survival; PSMA, prostate‐specific membrane antigen.

### 3.3 PSMA expression in the secondary brain tumours

**FIGURE 3:**
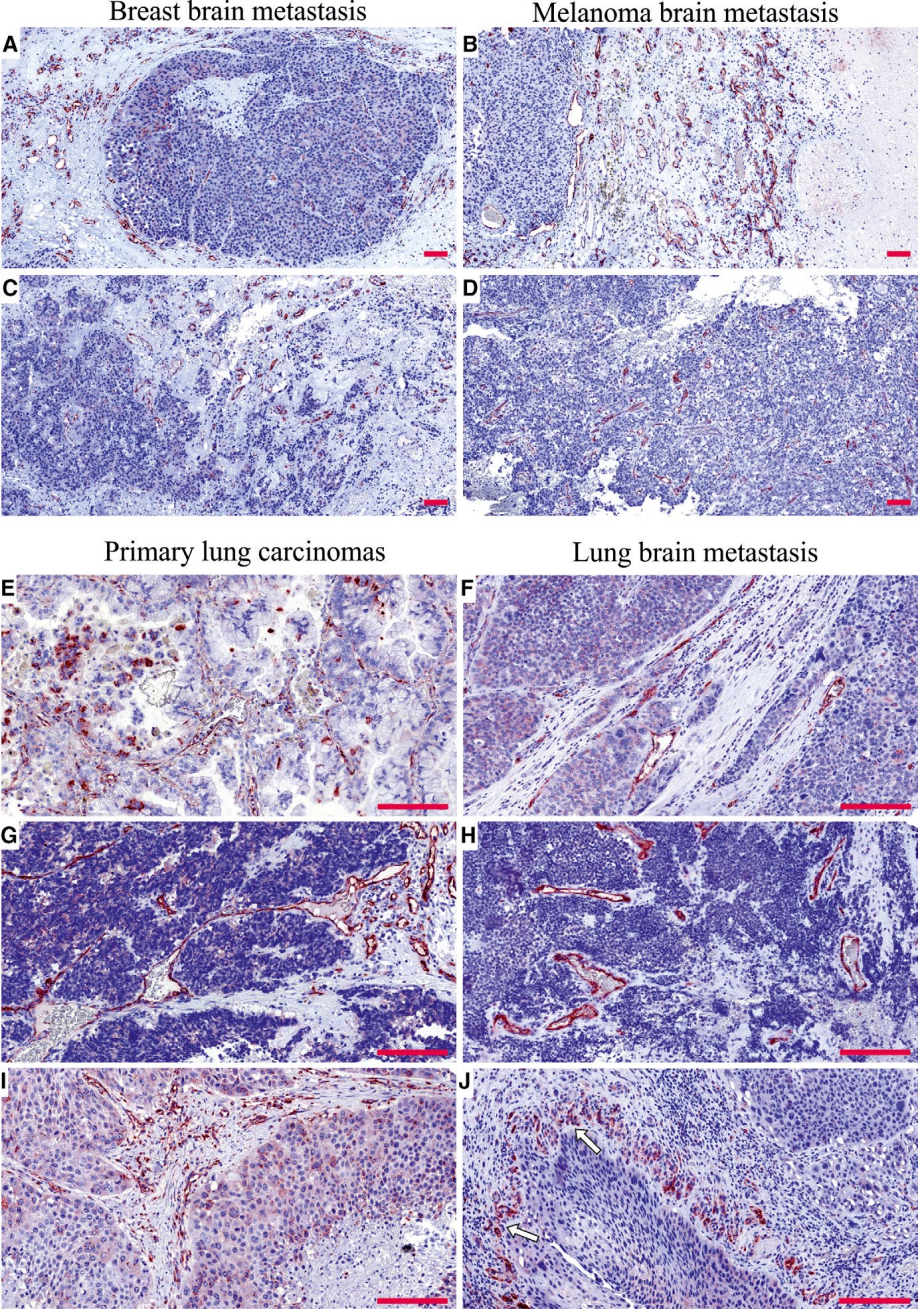
PSMA expression in the brain metastases of breast carcinomas and melanomas as well as in primary lung carcinomas and their associated metastases. A. PSMA is highly expressed in the vasculature of the brain metastases of breast adenocarcinoma (A) and invasive ductal carcinoma (C) as well as in the vasculature of melanoma brain metastases (B, D). E, G and I, Vasculature of the primary lung carcinomas of different histological subtype such as adenocarcinoma (E), small‐cell lung carcinoma (G) and squamous cell carcinoma (I) was highly positivity for PSMA. F, H and J, The brain metastases of the lung adenocarcinoma (F), small‐cell lung carcinoma (H) and squamous cell carcinoma (J) also expressed PSMA in their vasculature. There was a pattern of cellular pseudopalisades (arrows in J) closely surrounded by the PSMA‐positive vasculature similar to the one seen in glioblastomas. Scale bar = 100 μm. PSMA, prostate‐specific membrane antigen

**FIGURE 4:**
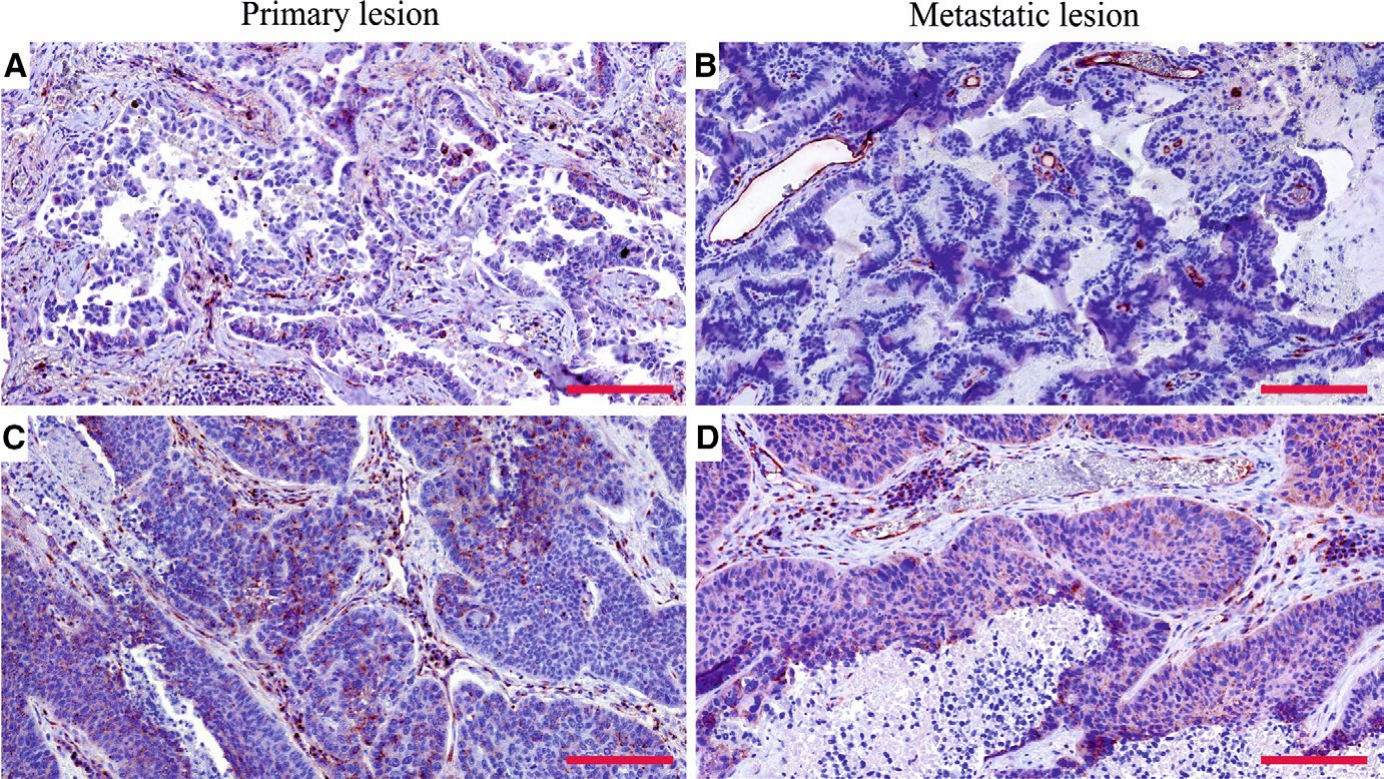
PSMA expression in the paired primary lung cancers and brain metastases from the same patient. A and B, PSMA expression in matched lung adenocarcinoma and brain metastasis. C and D, PSMA expression in matched lung squamous cell carcinoma and brain metastasis. Both the primary and metastatic tumour vasculature showed positivity for PSMA. Scale bar = 100 μm. PSMA, prostate‐specific membrane antigen

Extending our study, we analysed PSMA expression in the BMs of breast and lung carcinomas and melanomas. Of the whole tissue sections analysed, all 18/18 breast cancer BM samples (breast adenocarcinoma, n = 1; infiltrative duct carcinomas, n = 17) (Figure 3A,C) and 18/19 melanoma BMs (Figure 3B,D) expressed PSMA in their vasculature. Next, we compared PSMA expression in the primary lung tumours and their associated BMs and scored separately the vascular and tumour cell expression. The primary lung carcinoma samples in our tissue array were of various histological types containing (a) non‐small‐cell lung cancers such as adenocarcinomas and squamous cell carcinomas and (b) small‐cell lung carcinomas (SCLC). PSMA was expressed in the vasculature of all types of lung carcinomas (Figure 3E,G,I) and their metastases (Figure 3F,H,J and Table 4). Overall, the vascular expression of PSMA was highest in the lung cancer BMs (Figure 3F,H,J) and lowest in the melanoma BMs (Figure 3B,D), while the breast cancer BMs expressed moderate levels of PSMA (Figure 3A,C) as judged by visual evaluation. We then compared PSMA expression in the primary lung carcinomas and their BMs from the same patients (n = 52). In addition to the vascular expression of PSMA, the primary lung carcinomas (Figure 4A,C) and their corresponding metastatic lesions (Figure 4B,D) also expressed PSMA in the tumour cells. PSMA expression in the vasculature of the primary lung carcinomas and their metastatic lesions had no significant association with various clinical factors (histopathological staging, age or gender) (Table 5). As smoking is one of the major causative factors in lung tumours, patients were divided into three categories based on their smoking history: (a) no prior smoking, (b) stopped smoking and (c) still a smoker. No association between smoking and either the vascular or the cellular expression of PSMA was detected in primary lung carcinomas or their BMs (Table 5).

**TABLE 4:** Histological classification of PSMA‐positive tumours

Tumour		Tumour histology				
		Adenocarcinoma	Neuroendocrine	SCC	SCLC	Others
Lung carcinomas	Vascular	47.5% (19/40)	50% (1/2)	57.1% (4/7)	50% (2/4)	50% (1/2)
	Tumour cells	62.5% (25/40)	50% (1/2)	85.7% (6/7)	50% (2/4)	100% (2/2)
Lung BMs	Vascular	54.8% (23/42)	100% (2/2)	83.3% (5/6)	100% (4/4)	100% (2/2)
	Tumour cells	71.4% (30/42)	100% (2/2)	100% (6/6)	100% (4/4)	100% (2/2)

Abbreviations: BM, brain metastases; PSMA, prostate‐specific membrane antigen; SCC, squamous cell carcinomas; SCLC, small‐cell lung carcinomas.

**TABLE 5:** Clinicopathologic features of the primary lung carcinomas and their brain metastases

Pathological parameter		Features	Primary lung carcinomas				Lung brain metastasis			
			PSMA		Total	*P*‐value chi‐square	PSMA		Total	*P*‐value chi‐square
			+	−			+	−		
Pathological staging	I‐II	Vascular	15	18	33	*.50*	20	13	33	*.57*
	III‐IV		11	9	20		13	6	19	
	I‐II	Tumour cells	22	11	33	*.90*	24	9	33	*.29*
	III‐IV		13	7	20		17	2	19	
Gender	Male	Vascular	14	15	29	*.90*	18	11	29	*.72*
	Female		13	13	26		18	9	27	
	Male	Tumour cells	20	9	29	*.56*	22	7	29	*.61*
	Female		16	10	26		22	5	27	
Age	Min	Vascular	38	43	Median	*.57*	38	43	Median	*.19*
	Max		77	71	60^a^/57^b^		73	71	58^a^/62^b^	
	Min	Tumour cells	38	43	Median	*.93*	38	43	Median	*.13*
	Max		77	71	59^a^/56^b^		73	71	58^a^/63^b^	
Smoking	0‐1^c^	Vascular	10	10	20	*.92*	11	5	16	*.83*
	2^d^		16	17	33		25	13	38	
	0‐1^c^	Tumour cells	12	8	20	*.62*	12	4	16	*.71*
	2^d^		*22*	*11*	*33*		*31*	*7*	*38*	

Abbreviation: PSMA, prostate‐specific membrane antigen.

^(0)^Quit smoking.

^a^ PSMA‐positive.

^b^ PSMA‐negative.

^c^ Never smoked.

^d^ Still smoking^(1), (2)^.

### 3.4 Tumour cell expression of PSMA is higher in secondary than in primary brain tumours

**FIGURE 5:**
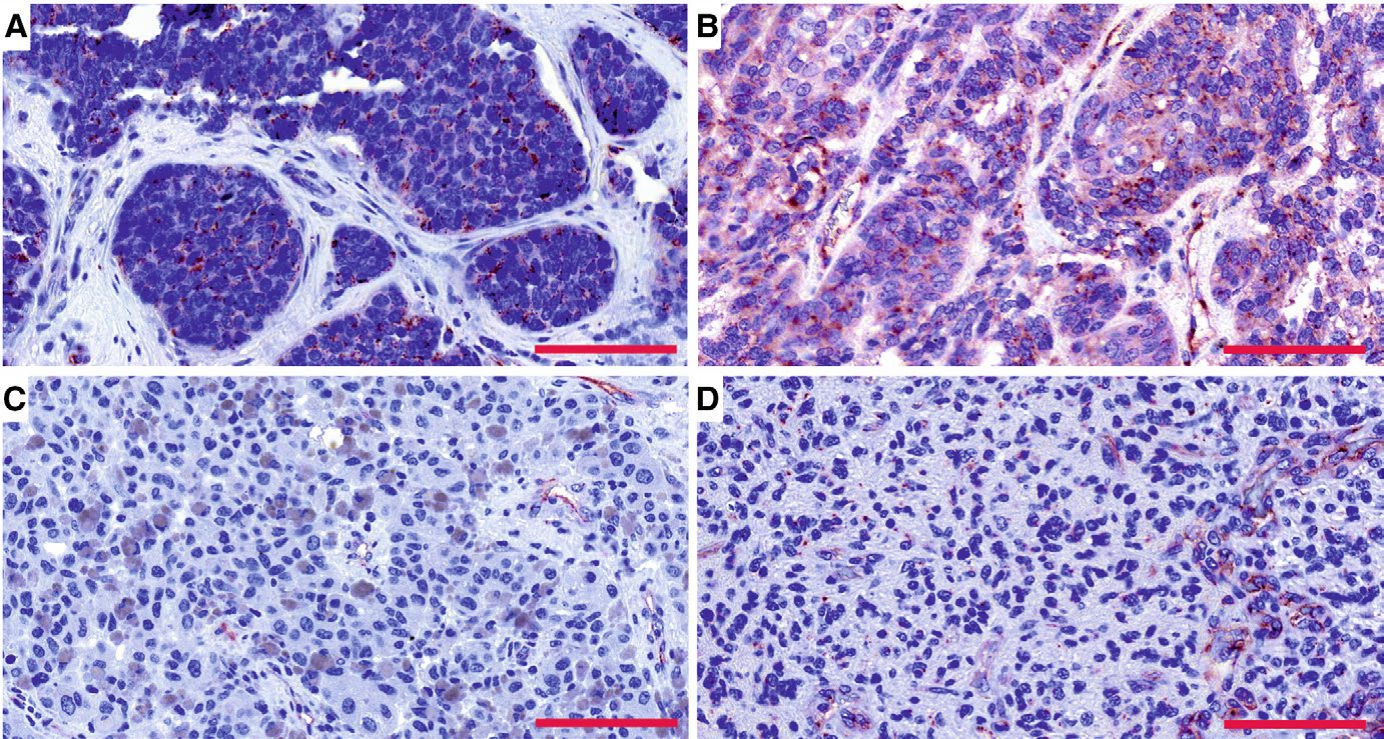
PSMA expression in the tumour cells varied between the tumour types. Amongst the primary and secondary brain tumours, the brain metastases of breast (A) and the lung (B) carcinomas showed the highest expression whereas the melanoma BMs (C) and glioblastomas (D) showed negligible expression of PSMA in the tumour cells. Scale bar = 100 μm. BM, brain metastases. PSMA, prostate‐specific membrane antigen

The tumour cell expression of PSMA varied substantially amongst the tumour types. When we compared PSMA expression between BMs of different origin, PSMA expression in tumour cells was higher in the BMs of lung and breast carcinoma than in melanoma BMs (Figure 5A‐C). The cellular expression of PSMA was very low in gliomas (Figure 5D), which is consistent with the Ivy GAP data set results (Figure 2B). In addition, tumour cell expression of PSMA in the lung carcinomas and their metastatic lesions did not associate with any clinical or molecular pathological parameters (Table 5).

### 3.5 Vascular PSMA expression in primary lung carcinomas was associated with significantly accelerated metastatic dissemination into brain

**FIGURE 6:**
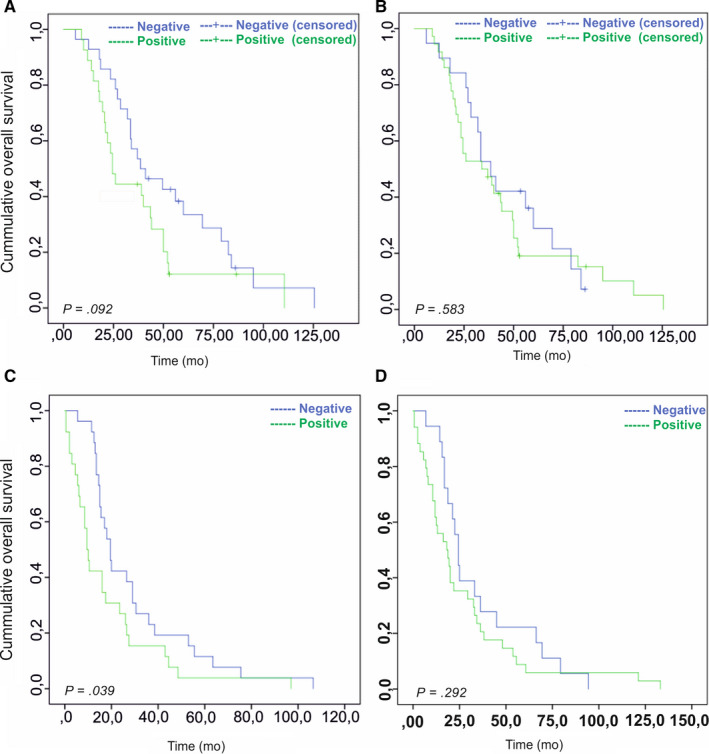
Association of PSMA expression with patient survival. A, The overall survival of the lung cancer patients (n = 55) whose tumours expressed PSMA in the vasculature (Mantel‐Cox *P* = 0.092/Breslow *P* = 0.051). B, The overall survival of the lung cancer patients (n = 55) with PSMA expression in tumour cells (Mantel‐Cox *P* = 0.583/Breslow *P* = 0.051). C, Primary lung carcinomas with vascular PSMA expression were associated with significantly accelerated rate of metastatic dissemination to brain (n = 52, Mantel‐Cox *P* = 0.039/Breslow *P* = 0.005). D, No significant association between brain metastases occurrence and PSMA expression in tumour cells was detected (n = 52, Mantel‐Cox *P* = 0.292/Breslow *P* = 0.074). PSMA, prostate‐specific membrane antigen

The overall survival data were known for 55 lung carcinoma patients. The vascular PSMA expression in the primary lung carcinomas was associated with a tendency towards decreased overall survival (Mantel‐Cox *P* = 0.092/Breslow *P* = 0.051) (Table 6, Figure 6A), whereas no association of PSMA expression in the tumour cells with the overall survival was observed (Mantel‐Cox *P* = 0.583/Breslow *P* = 0.33) (Figure 6B). In addition, PSMA expression either in the tumour vasculature or in tumour cells did not associate with the progression‐free survival (Table 6). Our patient cohort consisted of 52 matched primary lung carcinomas and their corresponding BMs. Primary tumours with PSMA‐positive vasculature were associated with significantly faster metastatic dissemination to the brain compared to tumours with PSMA‐negative vasculature (Mantel‐Cox *P* = 0.039/Breslow *P* = 0.005) (Table 7, Figure 6C). No association between PSMA expression in the tumour cells and accelerated metastatic dissemination was observed (Mantel‐Cox *P* = 0.292/Breslow *P* = 0.074) (Table 7, Figure 6D). The patients excluded from the analysis (n = 3) based on the diagnosis of the metastasis prior to the primary tumour detection still showed accelerated metastatic dissemination to the brain only if the vasculature was PSMA‐positive (Mantel‐Cox *P* = 0.012/Breslow *P* = 0.009) (Table S1, Figure S3A) but not the tumour cells (Mantel‐Cox *P* = 0.135/Breslow *P* = 0.046) (Table S1, Figure S3B).

**TABLE 6:** Overall survival and progression‐free survival based on PSMA expression

Tumour	Features	Function	PSMA (+) Total/censored	PSMA (−) Total/censored	Total Total/censored	*P*‐value Log‐rank/Breslow
Primary lung carcinomas	Vascular	OS	27/3	28/4	55/7	**.09/.05**
		PFS	27/18	28/16	55/34	.88/.80
	Tumour cells	OS	36/4	19/3	55/7	.58/.33
		PFS	36/23	19/11	55/34	.83/.79
Lung BMs	Vascular	OS	36/8	20/1	56/9	**.03/.01**
		PFS	36/23	20/11	56/34	.26/.24
	Tumour cells	OS	44/9	12/0	56/9	.13/.03
		PFS	44/26	12/8	56/34	.84/.67

Abbreviations: BMs, brain metastases; OS, overall survival; PFS, progression‐free survival; PSMA, prostate‐specific membrane antigen.

**TABLE 7:** Time to metastatic dissemination from the primary tumour to the brain (n = 52)

Tumour	Features	PSMA (+) Total/censored	PSMA (−) Total/censored	Total Total/censored	*P*‐Value Log‐rank/Breslow
Primary lung tumour	Vascular	26/0	26/0	52/0	**0.039/0.005**
	Tumour cells	34/0	18/0	52/0	0.292/0.074

Abbreviation: PSMA, prostate‐specific membrane antigen.

## 4 DISCUSSION

In this report, we studied the expression of PSMA in large cohorts of primary and secondary brain tumours, using both traditional clinical and molecular pathological methods. PSMA expression has been reported previously in gliomas,^15^ breast BMs^20^, ^21^ and primary lung carcinomas^22^ but with a very limited number of patients. Our study spans across diverse tumour types with a large set of patient samples including gliomas (n = 371) and BM of tumours that commonly metastasize to the brain such as breast BMs (n = 18), melanoma BMs (n = 19) and lung BMs (n = 52, with matched primary lung carcinomas). We assessed PSMA expression at the protein level using tumour TMA and associated it with various clinical and molecular pathological parameters following the WHO 2016 guidelines for the classification of tumours of the central nervous system. We also took advantage of the cancer genome databases such as the TCGA (n = 567) and Ivy GAP (n = 10) to assess PSMA expression at the RNA level.

Our results show that PSMA expression was highest in the vasculature of glioblastomas especially in the proliferating hyperplastic microvasculature, one of the key angiogenic and histopathological traits of glioblastomas. This result is in agreement with an earlier study that consisted of 14 glioma and 5 glioblastoma specimens.^15^ The study by Nomura et al^15^ showed moderate PSMA expression in grade I pilocytic astrocytomas (5 patients) and high expression in grade IV glioblastomas (GBM—5 patients), while grade II diffuse (4 patients) and grade III anaplastic (5 patients) astrocytomas were negative. The discrepancy between our study and Nomura et al^15^ may be explained by the relatively small number of patients in each glioma grade in the Nomura study. They also pointed out that the variability of PSMA staining (both vascular and tumour cells) within gliomas needs to be explored in larger scale studies, which we have now addressed here.

Microvascular hyperplasia is characterized by tufted micro‐aggregates, neovascular bodies with abundant and swiftly dividing endothelial cells, and inadequate pericyte/smooth muscle cell coverage. Microvascular hyperplasia, an essential feature associated with the tumour aggressiveness, is a potent predictor of poor prognosis.^23^ Our TMA results corroborated with the results we obtained using the Ivy GAP data set. The significance of the Ivy GAP project is the use of laser microdissection to sample individual anatomic structures of the tumour followed by RNA sequencing. This results in structure‐specific gene expression signatures, unlike the conventional tumour RNA sequencing in which all different anatomic structures and cell types are pooled together. We also show that integrin β1 (*ITGB1*) is up‐regulated in the PM. This is in concordance with the previous experimental report that showed PSMA‐dependent increase in integrin β1‐mediated signal transduction in a laminin‐specific manner to regulate angiogenic endothelial adhesion and invasion.^16^ It has been reported that the vascular expression of PSMA in squamous cell carcinoma of the oral cavity is associated with poor prognosis.^24^ In accordance with this, we show that vascular PSMA expression in gliomas associates with poor overall survival.

There are ample studies utilizing PSMA‐based imaging agents for tumour detection in prostate cancer,^25^, ^26^, ^27^, ^28^, ^29^, ^30^, ^31^, ^32^ high‐grade gliomas^33^, ^34^, ^35^, ^36^, ^37^ and lung BMs,^34^ as well as in follicular thyroid adenoma,^38^ metastatic renal cell carcinoma^39^ and melanoma and small‐cell lung cancer (SCLC) xenografts in vivo.^40^ However, recent reports showed that the cerebral radionecrotic uptake^41^ or stroke^42^ resulted in false‐positive diagnoses of cerebral metastases based on PSMA/CT uptake as limitation of the PSMA‐based diagnostic glioma imaging. A single‐arm phase II study where PSMA‐recognizing antibody‐drug conjugate (ADC) was used in progressive GBM patients (n = 6) following prior treatment with radiation, temozolomide and bevacizumab showed no demonstrable activity in these patients (NCT01856933). This was likely because of the minimal expression of the PSMA target and associated with dose‐limiting toxicity.^43^ In our study, the vascular expression of PSMA was observed in about 1/3 of glioblastomas. It thus raises a question of whether PSMA‐based targeting agents could be used in these patients after patient stratification.

In addition to the glioma samples, we screened PSMA expression in the BMs of lung and breast cancers and melanomas. Even though PSMA expression has been reported earlier in breast cancer BM,^15^, ^34^ we show that PSMA expression associates with the accelerated metastatic dissemination to the brain only when it is expressed by the vasculature of primary tumours and not by the tumour cells. The tendency towards decreased overall survival of patients expressing PSMA in the vasculature of their lung tumours may be used as a prognostic factor but needs to be further validated in a larger data set of patient samples. Future studies with a larger cohort of patient material and mechanisms of how PSMA in the tumour endothelial cells accelerates metastatic dissemination to brain would provide novel information about PSMA role in metastatic dissemination.

Prostate‐specific membrane antigen expression in the PM of glioblastoma near the CP is most likely a result of the hypoxia‐induced angiogenesis. However, the vascular expression in primary lung neoplasm leading to accelerated metastatic dissemination has not been described earlier. Interestingly, a very recent preclinical study of a similar protein to PSMA, the prostate‐specific antigen (PSA), could provide some clues on the molecular mechanism responsible for the accelerated metastasis.^44^ In this study, the authors show how PSA activates the vascular endothelial growth factor C (via a proteolytic cleavage) which in turn promotes a pro‐metastatic lymph/angiogenic niche.

In conclusion, we report that PSMA/*FOLH1* expression in the vasculature leads to poor patient outcome using large patient data sets as well as the genomic data sets. In the future, it will be interesting to see whether PSMA could be used as a diagnostic biomarker to detect and/or anticipate metastatic dissemination to the brain.

## CONFLICT OF INTEREST

The authors confirm that there are no conflicts of interest.

## AUTHORS’ CONTRIBUTIONS

JTR, VLJ and PL conceived and designed the study and wrote the manuscript. JTR performed the experiments and conceived the computational study. SL conceived and performed the computational study. HH and HS performed the statistical analysis. JT, HH, LR, VT and JM collected the samples and performed the pathological analyses. JT, MN, VLJ and PL supervised the study.

## INFORMED CONSENT

Patients included in the study have provided the informed consent.

## Supporting information

Table S1‐Figs S1‐S3

## Data Availability

The data that support the findings of this study are available from the corresponding author upon reasonable request.
