# Supplementary material for: Prostate‐specific membrane antigen expression in the vasculature of primary lung carcinomas associates with faster metastatic dissemination to the brain
Source: J Cell Mol Med. 2020 May 11;24(12):6916–27. doi: 10.1111/jcmm.15350 (PMC7299712; doi:10.1111/jcmm.15350)
Supplement: Supplementary file 1 — Table S1‐Figs S1‐S3 [file JCMM-24-6916-s001.pdf]

Supplementary material

Prostate Specific Membrane Antigen expression in the vasculature of primary lung carcinomas associates with faster metastatic dissemination to brain

Jayendrakishore Tanjore Ramanathan[1], Suvi Lehtipuro[2], Harri Sihto[1,3], József Tóvári[4], Lilla Reiniger [5,6], Vanda Téglási[6], Judit Moldvay[5,7], Matti Nykter[2,8], Hannu Haapasalo[9], Vadim Le Joncour[1] and Pirjo Laakkonen[1,10]

[1]Translational Cancer Medicine Research Program, Faculty of Medicine, University of Helsinki, Finland

[2]Faculty of Medicine and Health Technology, Tampere University, Tampere, Finland

[3]Clinicum, Faculty of Medicine, University of Helsinki, Finland

[4]Department of Experimental Pharmacology, National Institute of Oncology, Budapest, Hungary

[5]SE-NAP Brain Metastasis Research group, 2nd Department of Pathology, Semmelweis University, Budapest, Hungary

[6]1st Department of Pathology and Experimental Cancer Research, Semmelweis University, Budapest, Hungary

[7]Department of Tumor Biology, National Korányi Institute of Pulmonology–Semmelweis University, Budapest, Hungary

[8]Science Center, Tampere University Hospital, Tampere, Finland

[9]Department of Pathology, University of Tampere and Fimlab laboratories, Tampere, Finland.

[10]Laboratory Animal Centre, HiLIFE – Helsinki Institute of Life Science, University of Helsinki, Finland

Corresponding author: Prof. Pirjo Laakkonen, Translational Cancer Medicine Research Program, Haartmaninkatu 8, 00014 University of Helsinki, Finland.

Running title: Vascular PSMA accelerates brain metastasis

Table S1. Time to metastatic dissemination from the primary tumor to the brain (n = 49).

Tumor	***Features***	***PSMA (+)*** ***Total/censored***	***PSMA (-)*** ***Total/censored***	***Total*** ***Total/censored***	***P-Value*** ***Log-rank/Breslow***
**Primary lung tumor**	Vascular	24/0	25/0	49/0	**0.012/0.009**
	Tumor cells	31/0	18/0	49/0	0.135/0.046

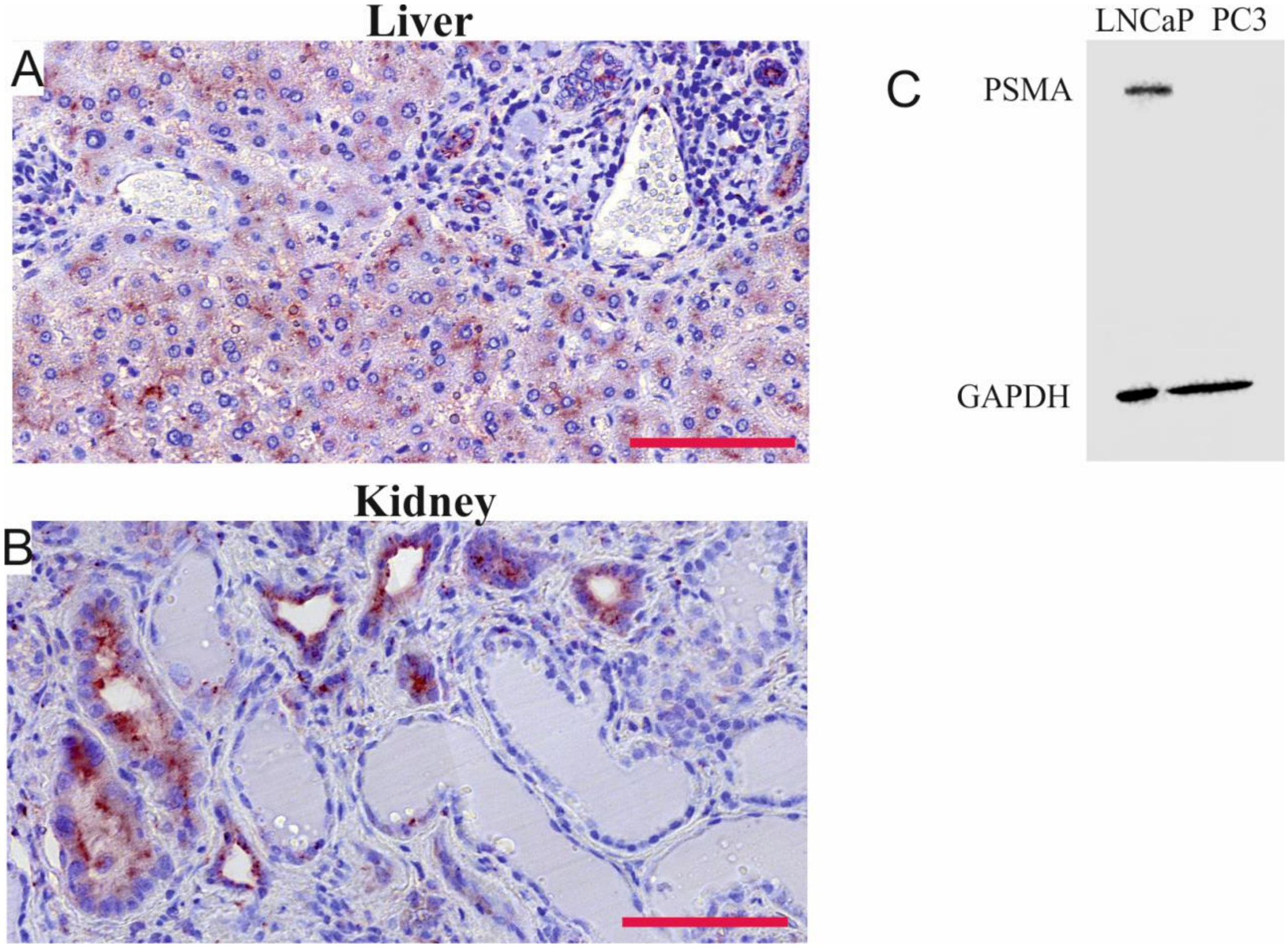

Supplementary Figure S1. A. Evaluation of the PSMA immunostaining**.** The normal human liver (A) and kidney (B, renal tubules) showed expression of PSMA (red color). C. The anti-PSMA antibody specifically recognizes PSMA as visualized by the presence of a single band in the PSMA positive LNCaP prostate cancer cell line versus the absence of signal in the PSMA negative PC3 prostate cancer cell line even when double the amount of PC3 protein extract was loaded. Scale bar = 100 μm.

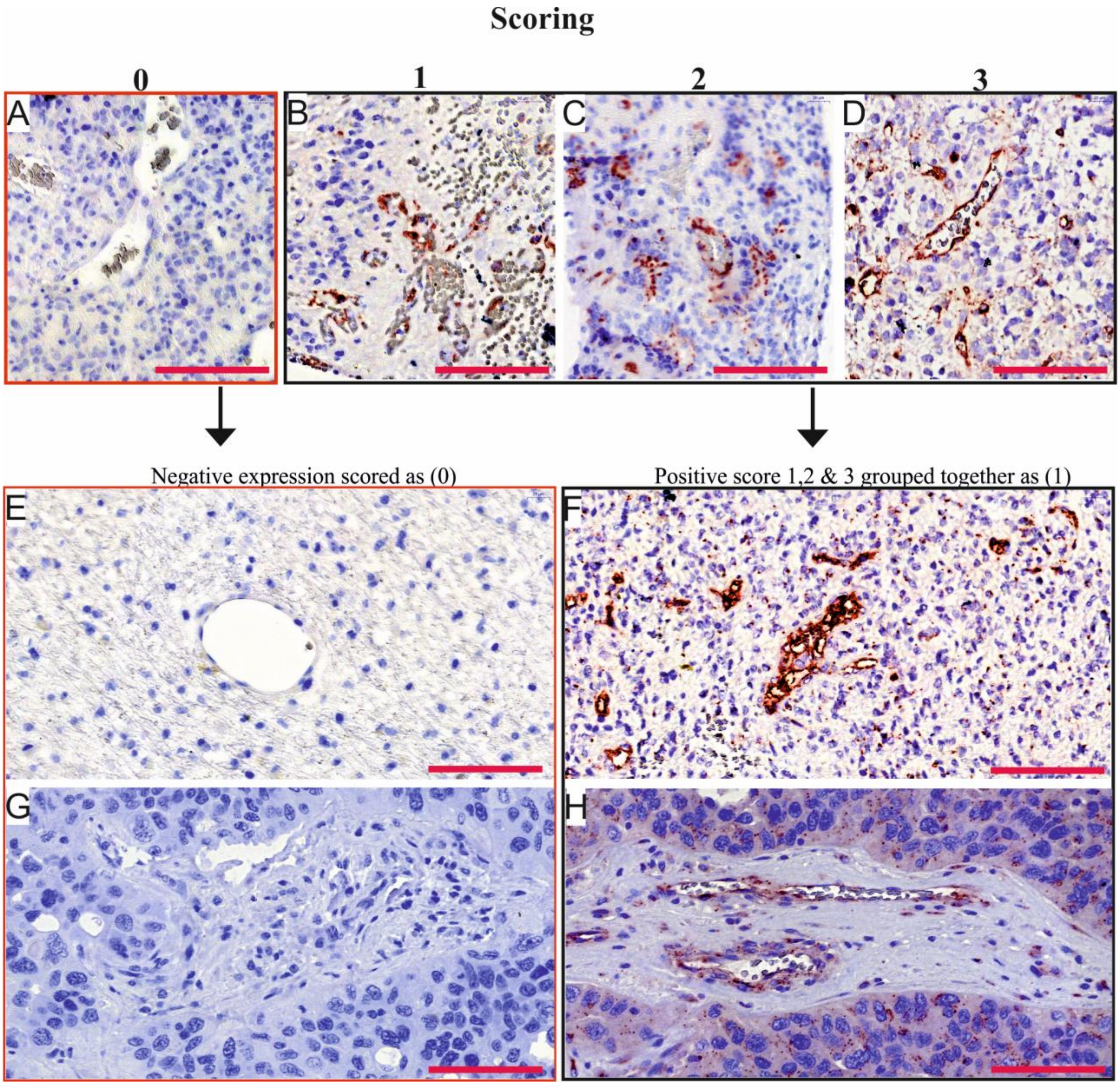

Supplementary Figure S2. The evaluation of the PSMA immunostaining**.** PSMA immunostaining (red) was scored was scored initially as negative (0) (A) or weakly (1, B), moderately (2, C) or highly (3, D) positive. We scored separately the vascular and tumor cell expression of PSMA and associated both separately with the clinical and molecular pathological parameters. In the analyses we and retained negative as (0) (E,G) and grouped all the positive scores as (1) (F,H). A-F, gliomas; G,H, lung carcinomas. Scale bar = 100 μm.

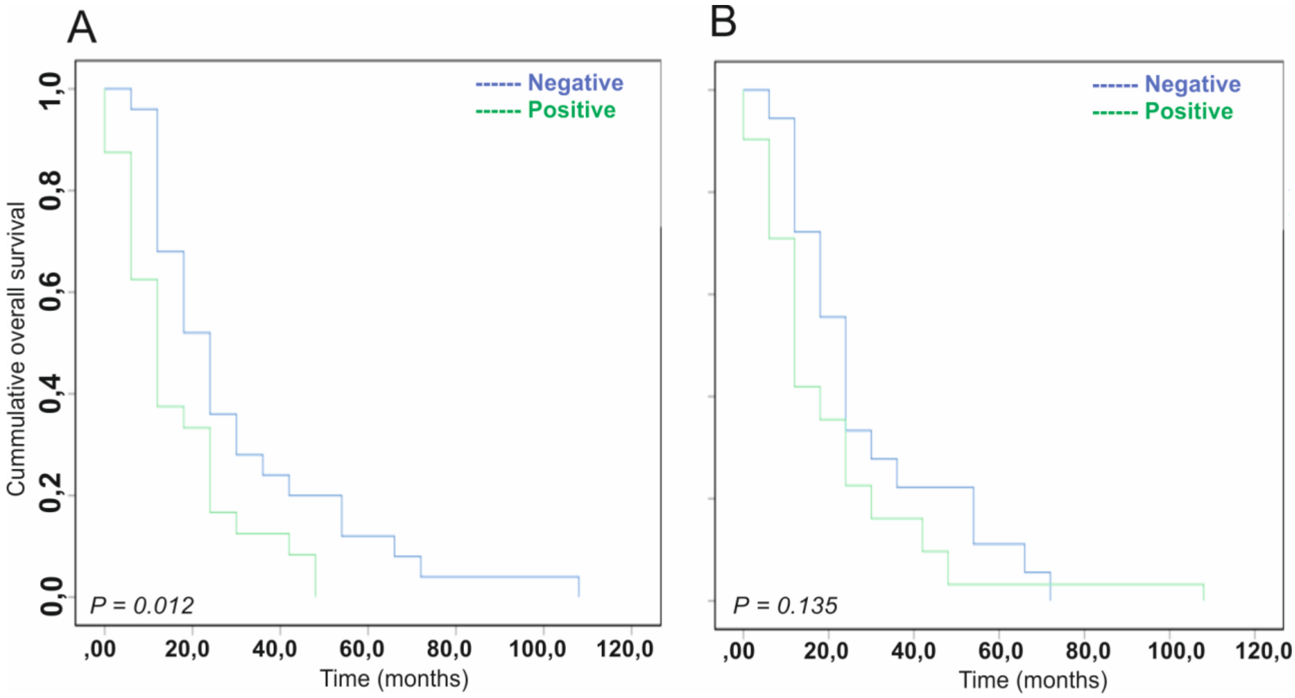

Supplementary Figure S3. Analysis of the accelerated metastatic dissemination to the brain after exclusion of three patients, due to the diagnosis of metastasis earlier than the primary tumor. The analysis still showed a distinct association with accelerated metastatic dissemination to the brain only if the vasculature was PSMA-positive (A, n = 49, Mantel-cox P = 0.012 / Breslow P = 0.009) but not when the tumor cells were positive **(**B, n = 49, Mantel-cox P = 0.135 / Breslow P = 0.046).

Supplementary methods

Cell culture

PSMA positive prostate cancer cell line LNCaP and PSMA negative prostate cell line PC3 (1-3) (gift from Dr. Hannu Koistinen, University of Helsinki) cells were maintained in RPMI 1640 media (Lonza) supplemented with L-glutamine (Lonza), 10% fetal bovine serum (GIBCO) and Penicillin/ streptomycin (Lonza) at 37°C

Western blot

The complete RIPA buffer (50mM Tris HCl pH 7.4, 150mM NaCl, 1% Sodium Deoxycholate, 1% SDS, 2% Octyl-β-D-Glucopyranoside with protease and phosphatase inhibitors) was used for lysis of cells. The supernatant was collected and Pierce™ BCA Protein Assay Kit (Thermo Scientific™, 23225) was utilized for determination of the protein concentration. The cell extracts were diluted in SDS laemmli loading buffer (supplemented with 5% β-mercaptoethanol), boiled for 5 minutes at

95°C and electrophoresed using NuPAGE Bis-Tris Gels, and then transferred onto PVDF membranes. The blocking was performed for a period of 60 minutes using TBS-T buffer supplemented with 5% non-fay dry milk. The primary rabbit anti-PSMA (ab133579, Abcam) and rabbit anti-GAPDH (2118S, Cell Signaling Technology), and the secondary antibodies (swine anti-rabbit-HRP polyclonal antiboy, (P0217) Dako) were diluted in blocking buffer and incubated at +4°C overnight and 1 hour at room temperature. The proteins were detected using the Pierce ECL western blotting substrate and imaged using LI-COR Odyssey Fc system.

References

1. Ghosh A, Wang X, Klein E, Heston WDW. Novel Role of Prostate-Specific Membrane Antigen in Suppressing Prostate Cancer Invasiveness. *Cancer Res.*2005;65(3):727-31.
2. Lee S, Roche P, Giannopoulos P, Mitmaker E, Tamilia M, Paliouras M, *et al.* Prostate-specific membrane antigen–directed nanoparticle targeting for extreme nearfield ablation of prostate cancer cells. *Tumor Biol.* 2017;39:101042831769594.
3. Gorges T, Riethdorf S, Ahsen O, Nastały P, Röck K, Boede M, *et al.* Heterogeneous PSMA expression on circulating tumor cells - a potential basis for stratification and monitoring of PSMA-directed therapies in prostate cancer. *Oncotarget.* 2016;7:4930-34941.
